# Physical Exercise or Cognitive Behavioral Therapy for Takotsubo Cardiomyopathy: A Randomized Controlled Trial

**DOI:** 10.1161/CIRCHEARTFAILURE.125.013229

**Published:** 2025-12-03

**Authors:** David T. Gamble, James Ross, Hilal Khan, Lesley Cheyne, Amelia Rudd, Janaki Srivanasan, Graham Horgan, Duncan Hogg, Phyo K. Myint, David E. Newby, Christopher Williams, Stuart R. Gray, Dana Dawson

**Affiliations:** 1 Cardiology Research Group, Aberdeen Cardiovascular and Diabetes Centre (D.T.G., H.K., L.C., A.R., J.S., D.H., P.K.M., D.D.), School of Medicine and Dentistry, University of Aberdeen, United Kingdom.; 2 Aberdeen Biomedical Imaging Centre (J.R.), School of Medicine and Dentistry, University of Aberdeen, United Kingdom.; 3 Biomathematics and Statistics Scotland, Aberdeen, United Kingdom (G.H.).; 4 BHF Centre for Cardiovascular Sciences, University of Edinburgh, United Kingdom (D.E.N.).; 5 School of Health and Wellbeing (C.W.), University of Glasgow, United Kingdom.; 6 School of Cardiovascular and Metabolic Health (S.R.G.), University of Glasgow, United Kingdom.

**Keywords:** cardiac rehabilitation, cognitive behavioral therapy, exercise, heart failure, Takotsubo cardiomyopathy

## Abstract

**BACKGROUND:**

Takotsubo cardiomyopathy is an acute cardiac emergency presenting with severe left ventricular dysfunction. Physical exercise training or cognitive behavioral therapy may enhance myocardial recovery after takotsubo cardiomyopathy.

**METHODS:**

In a prospective multicenter clinical trial conducted between February 2020 and August 2023, patients with acute takotsubo cardiomyopathy were randomized 1:1:1 to physical exercise training, cognitive behavioral therapy, or standard care for 12 weeks after index presentation. The primary end point was resting phosphocreatine/gamma-ATP ratio assessed by ^31^P-magnetic resonance spectroscopy. Secondary end points were the rate of oxygen consumption at peak exercise on cardiopulmonary exercise testing, 6-minute walk distance, left ventricular global longitudinal strain, and the Minnesota Living With Heart Failure Questionnaire. Twelve-week changes in outcome were compared between allocated trial interventions.

**RESULTS:**

Seventy-six participants were recruited: the median age was 66 years, and 91% were women. Compared with standard care, the primary end point of myocardial phosphocreatine/gamma-ATP ratio was improved by physical exercise training (0.4 [95% CI, 0.1–0.8]; *P*=0.016) and cognitive behavioral therapy (0.3 [0.01–0.7]; *P*=0.043). Both physical exercise training and cognitive behavioral therapy improved rate of oxygen consumption at peak exercise (4.7 [1.4–8.0] and 4.0 [1.5–6.4] mL/min per kg; *P*=0.001 and 0.004, respectively) and 6-minute walk distance (92.6 [24.7–160.6] and 73.3 [7.9–138.8] m; *P*=0.004 and 0.029, respectively) compared with standard care. There were no differences in global longitudinal strain or symptom burden.

**CONCLUSIONS:**

In patients with acute takotsubo cardiomyopathy, a 12-week intervention with exercise training or cognitive behavioral therapy improved left ventricular myocardial energetics and exercise performance without demonstrable effects on symptoms of heart failure.

**REGISTRATION:**

URL: https://www.clinicaltrials.gov; Unique identifier: NCT04425785.

**What is New?**

- In a clinical randomized controlled trial, 12-week rehabilitation with behavioral modification (physical exercise training or cognitive behavioral therapy) delivered to patients with acute takotsubo cardiomyopathy led to improvements in resting left ventricular high-energy phosphate metabolism and cardiorespiratory functional capacity.

**What Are the Clinical Implications?**

- Behavioral modification strategies after takotsubo cardiomyopathy can enhance the metabolic and functional recovery of the heart, supporting the use of these cardiac rehabilitation strategies for these patients.

**See Editorial by Upadhya and Kitzman**

Patients with takotsubo cardiomyopathy present acutely with symptoms indicative of an acute myocardial infarction. However, these patients often have unobstructed coronary arteries and severe left ventricular dysfunction with characteristic regional ballooning.^1^ Despite apparent rapid normalization of heart function, many patients develop heart failure with preserved ejection fraction, characterized by impaired cardiac energetic status and cardiac limitation on cardiopulmonary exercise testing (reduced rate of oxygen consumption at peak exercise [peak VO_2_] and increased slope of the minute ventilation/carbon dioxide production relationship).^2,3^ Indeed, overall survival and subsequent clinical outcomes are poor compared with age, sex, and comorbidity-matched populations, and approach those who sustain an acute myocardial infarction.^1,4^

Current management strategies focus on complications surrounding the acute presentation with no robust evidence available for medium or long-term improvement of symptoms, functional status, or survival outcomes.^5^ In heart failure with preserved ejection fraction or other causes, patients’ functional status can be improved with exercise training programs,^6^ but this has not been investigated in takotsubo cardiomyopathy. In addition, psychiatric disorders including anxiety or depression are more common in patients with takotsubo cardiomyopathy,^7^ suggesting that some patients might benefit from a combined psychocardiological rehabilitation. However, there are no randomized controlled trials to inform the management of patients with takotsubo cardiomyopathy, and the benefits of nonpharmacological intervention remain unknown in this patient population.

We have previously shown profound metabolic and energetic impairment in acute takotsubo syndrome, followed by a protracted and incomplete recovery.^2^ Impaired energetics often precede structural or functional decline, making it a sensitive early marker for disease progression. Studies show that reduced cardiac energy efficiency is associated with higher mortality and increased hospitalization rates for cardiac conditions of other causes.^8^

In this trial, we assessed 12-week cardiac rehabilitation interventions using either physical exercise training or cognitive behavioral therapy in patients with a recent presentation of acute takotsubo cardiomyopathy. The primary end point was resting left ventricular energetics status measured by ^31^P-magnetic resonance spectroscopy and the secondary end points of cardiovascular fitness (assessed by cardiopulmonary exercise testing and 6-minute walk distance), global left ventricular longitudinal strain on echocardiography, and patient-reported symptoms.

## Methods

Upon reasonable request, the authors will make the data, methods, and materials available to any researcher for purposes of reproducing the results or replicating the procedure without compromising patient confidentiality.

### Trial Design and Patient Population

This was a multicenter, prospective, randomized, open-label, blinded end point design trial. The study was approved by the institutional review board (North of Scotland Ethics Committee One), all participants provided informed consent, and the study was prospectively registered (NCT04425785). Patients were invited to participate if they met the contemporaneous diagnostic criteria for acute takotsubo cardiomyopathy.^9^ In brief, acute takotsubo cardiomyopathy was defined by (1) symptoms (chest pain, dyspnea, or collapse), (2) left ventricular wall motion abnormality showing apical, mid-cavity, or basal ballooning or focal dysfunction, (3) no culprit lesion on coronary angiography, and (4) elevation in plasma cardiac biomarkers (troponin). The presence of coronary artery disease or a normal ECG did not preclude the diagnosis. Patients were recruited within 3 weeks of the acute diagnosis. Exclusion criteria were physical limitations preventing a reasonable pursuit of a physical exercise program, pregnancy, contraindications to magnetic resonance scanning, or inability to attend the study visits. Recruitment was performed between February 5, 2020, and August 20, 2023 (paused during March 23 to July 2, 2020, due to the pandemic), at 6 Scottish hospitals: Aberdeen, Glasgow, Lanarkshire, Dundee, Edinburgh, and Inverness with all trial investigations being conducted at Aberdeen Royal Infirmary. Patients were randomized immediately after consent into 1 of 3 arms: (1) standard care, (2) physical exercise training, or (3) cognitive behavioral therapy, through a central computer-generated randomization algorithm (within blocks of 6) to ensure concealment of trial allocation.

### Trial Interventions

#### Physical Exercise Training

Participants undertook a 12-week exercise training program starting with 3×20 min of exercise in the first week, building progressively to 5×60 min of exercise after week 6 of the intervention until week 12 (Table 1 details the full training schedule). Typically, sessions included cycling/stepper/treadmill in the gym, locally provided classes (aerobics), or swimming. During the COVID-19 pandemic, stationary exercise bikes were provided and installed in the patients’ homes. During sessions, physical exertion was self-reported using a rating of perceived exertion scale^10^ for monitoring adherence and for gradually increasing from very light exertion at week 1 to moderate/somewhat hard exertion at week 12. Rating of perceived exertion is a validated method for prescribing exercise training intensity with well-documented correlation with physiological parameters, including heart rate, blood lactate concentration, maximal rate of oxygen uptake, and respiration rate.^11^ Participants were monitored remotely with at least weekly telephone calls. For participants who were subject to a protocol deviation due to the COVID-19 pandemic, they continued their exercise intervention at the week 12 level with remote monitoring at least weekly.

**Table 1.**
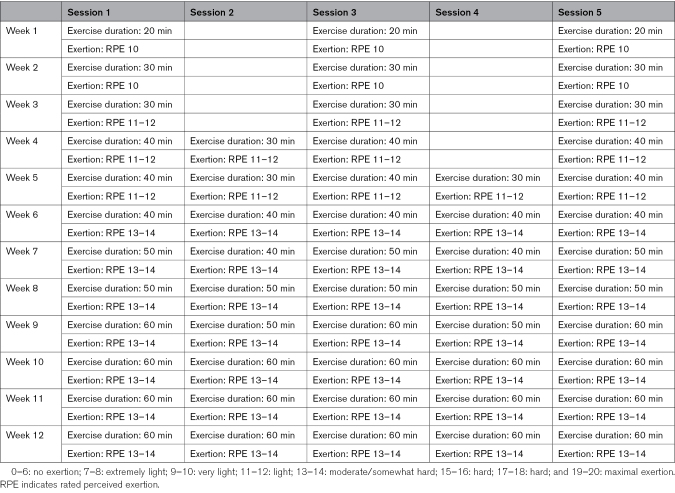
Physical Exercise Protocol

#### Cognitive Behavioral Therapy

This consisted of a series of 12 educational life skill sessions, purposefully adapted for sufferers of takotsubo cardiomyopathy using the original Living Life to the Full classes developed by a co-investigator of this trial (C.W.). The course was delivered on a one-to-one basis using an audiovisual platform by 2 physicians who had received appropriate training under the supervision of an accredited cognitive behavioral therapy practitioner and consultant psychiatrist (C.W.), plus weekly or daily support provided by the trial physicians (D.T.G. and H.K.). Participants were also provided with written information from the linked course booklets. The 12 sessions (Table 2) draw on psychoeducation, noticing and changing unhelpful thinking, building activities that provide pleasure, connection with others and a sense of achievement, problem solving, improving sleep, and more. These aimed to help participants understand why they feel as they do and then make focused changes to their thoughts, behavior, and relationships to improve mood, anxiety, and social function. Participants who were subject to a protocol deviation due to the COVID-19 pandemic continued their cognitive behavioral therapy intervention with weekly revision sessions. The cognitive behavioral therapy group did not receive information on exercise training.

**Table 2.**
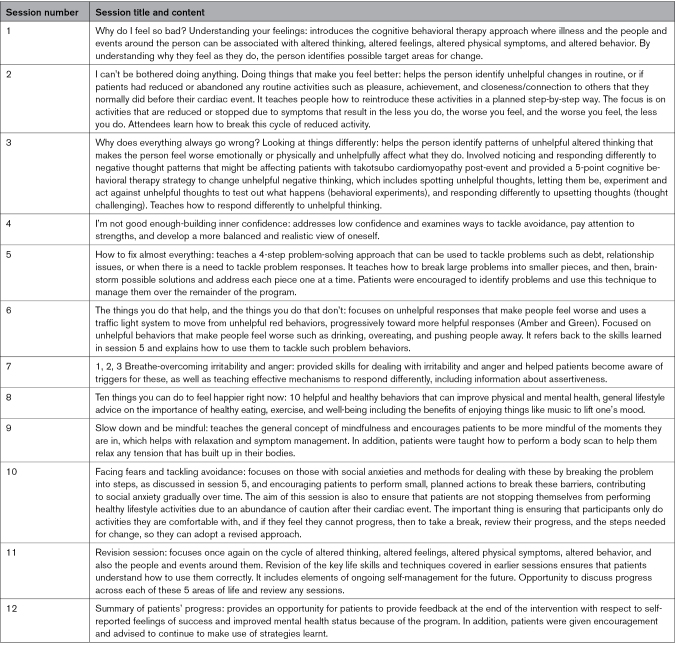
Cognitive Behavioral Therapy Session Titles and Content of the Living Life to the Full With Takotsubo Course

#### Standard Care

Patients were treated according to the practice of their attending physician. The standard of care group did not receive advice on exercise training or cardiac rehabilitation.

### Study Assessments

Clinical assessments were undertaken at baseline (within 3 weeks of diagnosis of acute takotsubo) and after delivery of the 12-week cardiac rehabilitation intervention. These assessments included ^31^P-magnetic resonance spectroscopy, cardiac magnetic resonance imaging, physical capacity assessments, transthoracic echocardiography, blood sampling, and completion of the Minnesota Living With Heart Failure Questionnaire (Figure S1 shows the study consort diagram). Patients enrolled during the national governmental public health restrictions imposed by the COVID-19 pandemic could not return after 12 weeks due to travel and hospital entry; thus, a protocol deviation was logged, and their interventions and follow-up were continued until the end of the study visit could be completed.

### ^31^Phosphorus Magnetic Resonance Spectroscopy and Cardiac Magnetic Resonance Imaging

All participants were scanned on a 3T Philips scanner (Achieva; Philips Medical Systems, Best, the Netherlands). ^31^P-magnetic resonance spectroscopy was acquired using a 14-cm diameter transmit-receive ^31^P-surface coil (Philips Healthcare, Best).^12^ Signal localization was performed using 1D-chemical shift imaging adiabatic excitation with participants in the supine position. For in vivo resting cardiac energetics, the phosphocreatine/gamma-ATP (γATP) ratio was determined after the γATP was corrected for blood contamination and saturation correction applied as described previously.^13^ Following this, a 32-channel phased array coil was used to acquire an ECG-gated cardiac magnetic resonance protocol inclusive of cine imaging, T1/T2 mapping, and late gadolinium enhancement. Cardiac magnetic resonance images were analyzed in Circle cvi42 v5.16 (Circle Cardiovascular Imaging, Calgary, AB, Canada) for deriving indexed left ventricular volumes and mass, and ejection fraction. T1 and T2 maps were analyzed with the Philips IntelliSpace software version 11.1 (Koninklijke Philips N.V., Amsterdam, the Netherlands).

#### Physical Exercise Capacity

Daytime activity levels were measured using ActiGraph wGT3X-BT activity monitors (ActiGraph, United States) and converted into physical activity metrics of minutes per day, which were considered sedentary, light, moderate, vigorous, and very vigorous activity using the Freedson cutoff ranges.^14^

A cardiopulmonary exercise test was performed on a treadmill (Cosmed T150DE Treadmill, Cosmed, Italy) integrated with a metabolic system (Quark PFT, Cosmed). A progressive incremental ramp protocol of 1 km/h every minute and a base elevation of 1% gradient was used until volitional exhaustion. Oxygen consumption (VO_2_) was measured breath by breath, and 12-lead ECG, heart and respiratory rate, blood pressure, and oxygen saturations were measured throughout. Any test with a respiratory exchange ratio <1.1 was classed as suboptimal and was excluded from the analysis. A standard 6-minute walk test^15^ on a flat, straight course, hard surface was performed using a Digital Distance Measuring Wheel to record the distance.

The 6-minute walk test was conducted on a straight, flat, course without pedestrian traffic at Aberdeen Royal Infirmary. Those using walking aids were instructed to use them, with usage documented. If a participant stopped, the timer continued, while they could rest.

#### Transthoracic Echocardiography

Two-dimensional and Doppler echocardiography was performed using a Vivid E9 system with a 2.5-MHz (M5S) transducer (GE Vingmed, Horten, Norway) by a British Society of Echocardiography–accredited sonographer, and image analysis was performed by 2 experienced operators (D.T.G. and J.S.) on the TOMTEC-ARENA v6 (Tomtec, Unterschleißheim, Germany). Three cine loops in each of the standard views (parasternal long-axis, short-axis, and apical 4-, 3-, and 2-chamber) were obtained at a frame rate of at least 85 Hz, together with color, and continuous and pulsed wave Doppler examinations were stored for offline analysis. Global longitudinal strain was obtained after manually adjusting the automatic detection of the epicardial and endocardial borders in each of the 3 long-axis views.

#### Cardiovascular Biomarkers

Blood samples were taken at rest from all participants, at peak exercise, and 15 minutes post-exercise in participants who underwent cardiopulmonary exercise testing. Serum was separated after centrifugation at 50*g* for 10 minutes and stored at −80 °C. Samples were analyzed for BNP (B-type natriuretic peptide) concentrations using an immunoassay (Alere Triage MeterPro, Delaware, USA). In patients with heart failure where cardiac limitation occurs during exercise and is not necessarily present at rest, BNP can increase rapidly after exercise due to raised left ventricular pressures and may indicate a higher severity heart failure phenotype. NT-proBNP (N-terminal pro-BNP) synthesis has a much longer half-life and is, therefore, more indicative of chronic heart failure.^16^ BNP has also been shown to negatively correlate with peak VO_2_ and, therefore, may be an effective way to monitor therapy and exercise programs in clinical settings where cardiopulmonary exercise testing is inappropriate or unfeasible.^17^

### Study End Points

The primary end point was the cardiac phosphocreatine/γATP ratio measured by ^31^P-magnetic resonance spectroscopy. Secondary end points included (1) physical capacity assessment (cardiopulmonary exercise test–derived peak VO_2_, slope of the minute ventilation/carbon dioxide production relationship, and 6-minute walk test distance); (2) global longitudinal strain from 2-dimensional echocardiography; and (3) questionnaire-derived symptom burden. Data for all end points were analyzed blinded to treatment allocation, in a random order at the end of the study.

### Statistical Analysis

Statistical analysis was performed using IBM SPSS Statistics, version 28.0. To detect the difference in change from baseline to follow-up of 0.4 (SD, 0.45) in the primary end point of phosphocreatine/γATP ratio, with 80% power and a 2-sided significance level of 5%, the sample size required 21 participants per group. Sample size calculations were based on previous work from our center on the assessment of phosphocreatine/γATP ratio as a gold standard for the appreciation of in vivo cardiac energetics in takotsubo cardiomyopathy and other cardiomyopathies.^3,18^ For baseline characteristics, differences in categorical data were assessed using binary logistic regression, a 1-way ANOVA for normally distributed data, and the Mann-Whitney *U* test for nonparametric data. Both the primary end point and secondary end point analyses were performed on a per-protocol set of patients with valid measurements available at baseline and post-intervention. Baseline demographic data are presented as mean and SD or number (%), unless stated otherwise. Responses to interventions are presented as the change from baseline to postintervention calculated for each participant by subtracting the baseline variable measurement from the postintervention measurement. Intervention arm effect was assessed with a general linear model followed by ANOVA with ordinary least squares for between-patient groups differences in the 12-week changes in outcomes with covariates for age, sex, and whether the intervention was affected by COVID-19. Post hoc comparisons between the 3 groups were based on the Fisher least significant difference. The regression table for this model is included in the Supplemental Methods as Table S1. Group comparisons were made using the least significant difference. Correlations were assessed using the Pearson correlation coefficient. Normality was checked for using the Shapiro-Wilk test, and data were analyzed using the Mann-Whitney *U* test if found to be not normally distributed. All data analysis was performed on anonymized data at the end of the study, and assessors were blinded to the group allocation of the participants. Individual missing data points were excluded, and no statistical methods to replace missing data were used.

## Results

Twenty-six patients with acute takotsubo cardiomyopathy were recruited into each of the cognitive behavioral therapy and physical exercise training groups and were compared with 24 patients with acute takotsubo cardiomyopathy in the standard care group. Patients had a median age of 66 (range, 38–79) years; 91% were female and had similar rates of comorbidities (Table 3). One-third of participants had ST-segment elevation on their ECG, and most had apical left ventricular ballooning. The median follow-up time was 113 (range, 81–211) days.

**Table 3.**
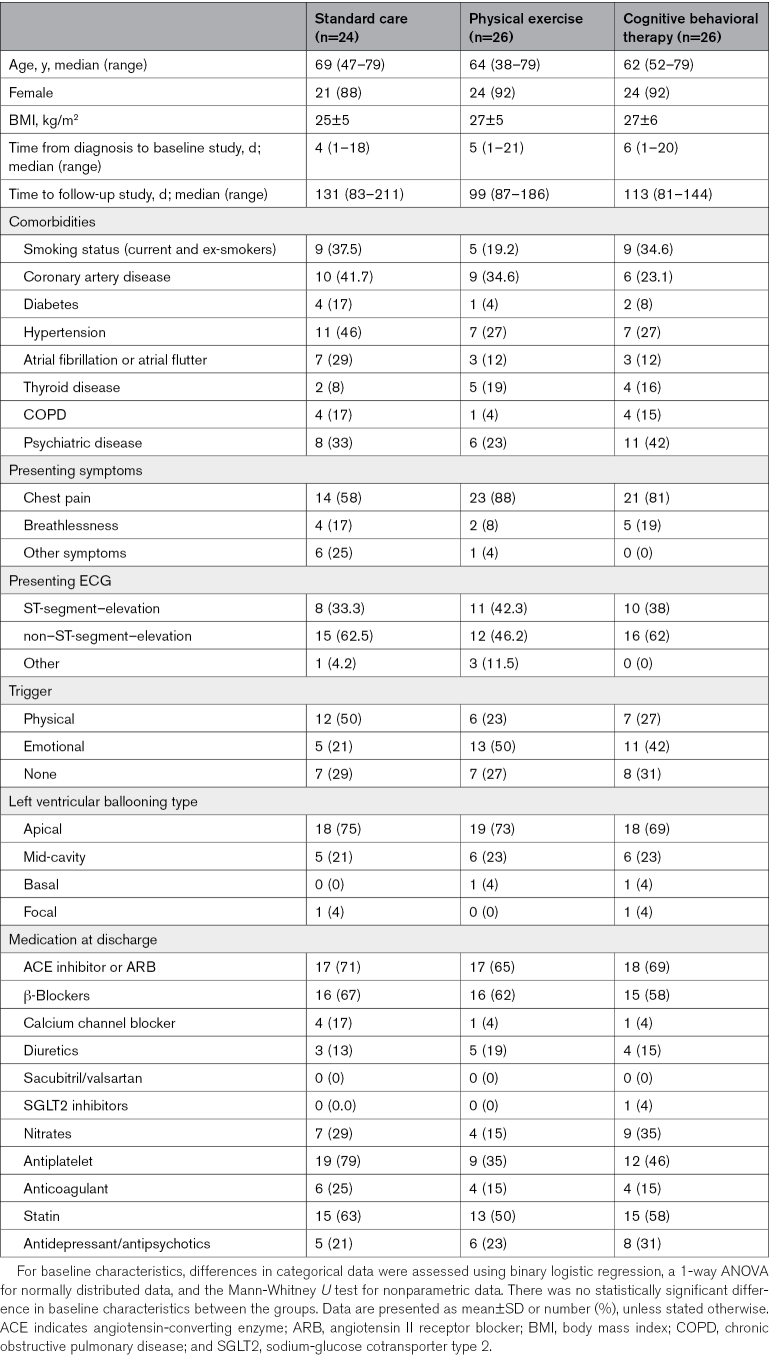
Baseline Characteristics, Past Medical History, Presenting ECG, Symptoms, Takotsubo Type, and Medications at Discharge of the Study Populations

Baseline cardiac ^31^P-magnetic resonance spectroscopy–derived phosphocreatine/γATP ratio, peak VO_2_, slope of the minute ventilation/carbon dioxide production relationship, 6-minute walk distance, echocardiography-derived global longitudinal strain, basal level of physical activity, and questionnaire scores were comparable between the 3 groups (Table 4).

**Table 4.**
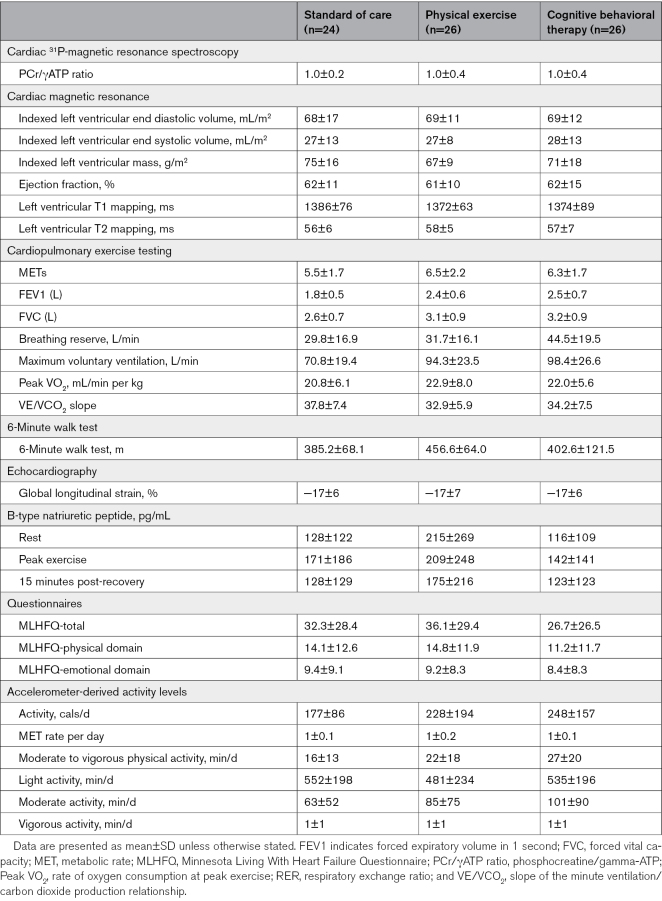
Study Assessments at Baseline

### Primary End Point

The resting myocardial energetic status measured by the phosphocreatine/γATP ratio improved the physical exercise group relative to the standard care group (0.4 [95% CI, 0.1–0.8]; *P*=0.016). Similar findings were apparent in the cognitive behavioral therapy group (0.3 [95% CI, 0.01–0.7]; *P*=0.043; Table 5; Figure).

**Table 5.**
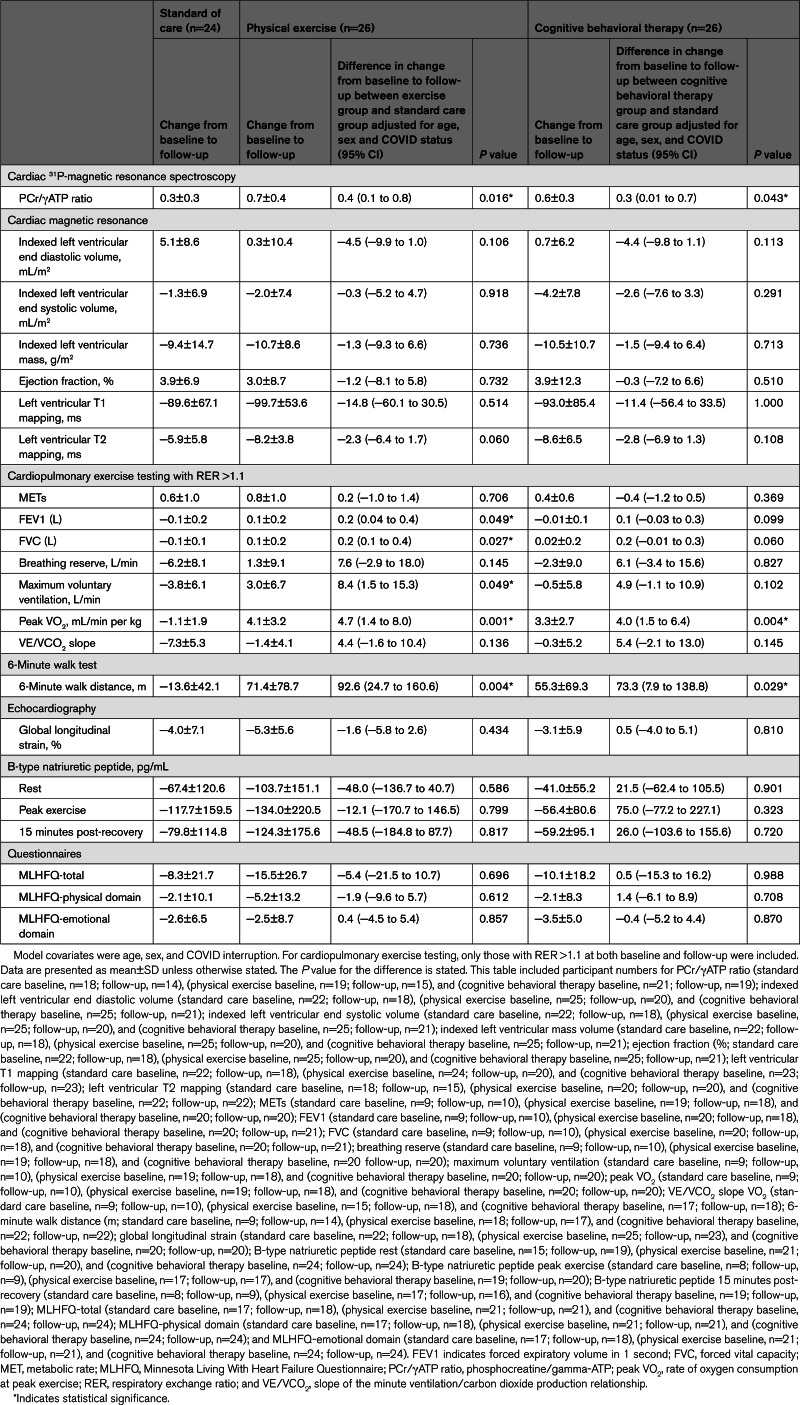
Comparison of Intervention Groups With Control Group

**Figure.**
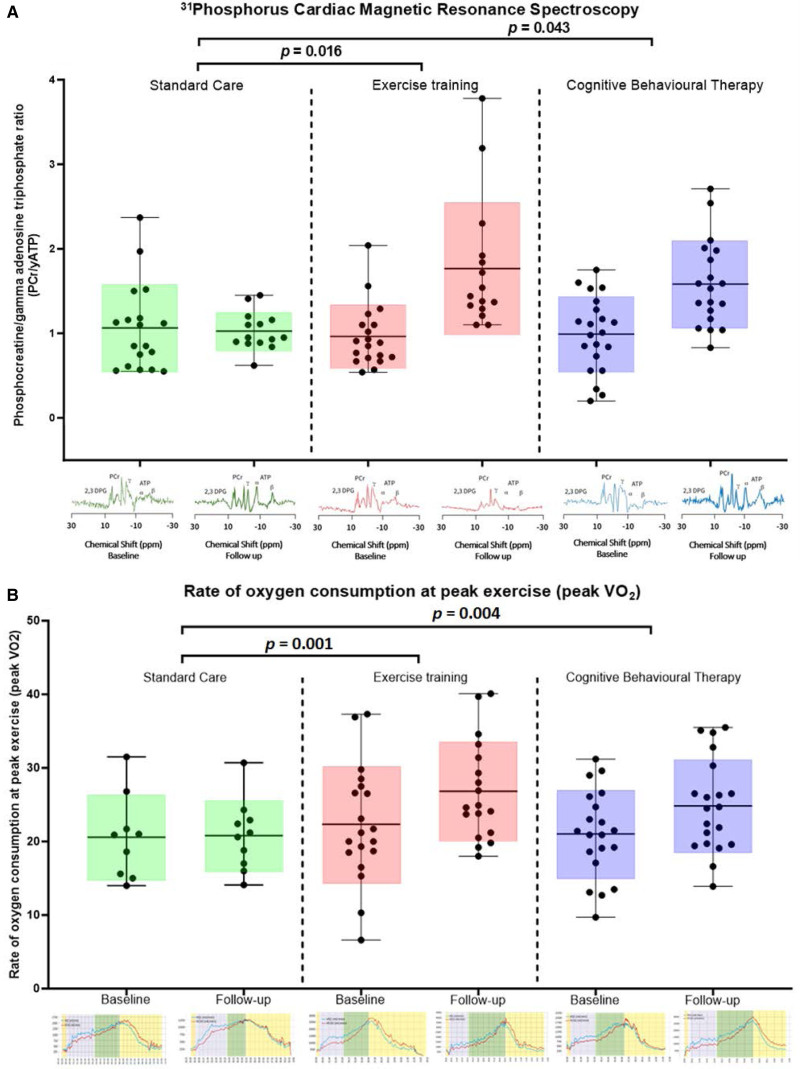
**Primary and secondary end points.** ^31^P-magnetic resonance spectroscopy for resting cardiac energetic status (**A**) and cardiopulmonary exercise testing–derived rate of oxygen consumption at peak exercise (**B**). Data shown as mean and box plots/error bars (SD/range) with individual data points for each intervention group (standard care, physical exercise training intervention, and cognitive behavioral therapy) at baseline and follow-up in patients who completed both visits. Below each graph in **A**, an individual example of cardiac spectroscopy showing phosphocreatine (PCr) γ, β, and α ATP, and 2,3-diphosphoglycerate peaks. Below each graph in **B**, individual examples of cardiopulmonary exercise testing tracings of absolute oxygen consumption (VO_2,_ blue line) and carbon dioxide production (VCO_2,_ red line). The *x* axis represents time, and the *y* axis represents the exchange of VO_2_ and VCO_2_ in mL/min. The gray areas are the warm-up period, the green areas represent the exercise duration, and the yellow areas are the recovery time. The crossover between the blue (VO_2_) and red (VCO_2_) lines represents a respiratory exchange ratio of 1. The *P* value for the difference in change from baseline to follow-up between the intervention group and the standard care group was adjusted for age, sex, and COVID status.

### Secondary End Points

As shown in Table 5 and the Figure, in the physical exercise training group, there were improvements in peak VO_2_ (4.7 [95% CI, 1.4–8.0] mL/min per kg; *P*=0.001), forced expiratory volume in 1 second (0.2 [95% CI, 0.04–0.4] L; *P*=0.049), forced vital capacity (0.2 [95% CI, 0.1–0.4] L; *P*=0.027), maximum voluntary ventilation (8.4 [95% CI, 1.5–15.3] L/min; *P*=0.049), and 6-minute walk distance (92.6 [95% CI, 24.7–160.6] m; *P*=0.004) compared with standard care.

In the cognitive behavioral therapy group, there were improvements in peak VO_2_ (4.0 [95% CI, 1.5–6.4] mL/min per kg; *P*=0.004) and 6-minute walk distance (73.3 [95% CI, 7.9–138.8] m; *P*=0.029) compared with standard care.

Compared with standard care, there were no differences between either physical exercise or cognitive behavioral therapy groups for the 2D echocardiography-derived global longitudinal strain or patient-reported symptoms from the Minnesota Living With Heart Failure Questionnaire. There was no correlation between patient-reported symptoms from the Minnesota Living With Heart Failure Questionnaire and phosphocreatine/γATP ratio (r^2^=0.19; *P*=0.297), peak VO_2_ (r^2^=0.021; *P*=0.901), or 6-minute walk distance (r^2^=0.13; *P*=0.425).

Compared with the standard care group, there were no differences in either trial intervention group in other cardiac magnetic resonance measures (left ventricular end diastolic or end systolic volumes, left ventricular mass, ejection fraction, and T1 or T2 mapping) or cardiac biomarkers (BNP) at rest or after exercise.

## Discussion

This is the first randomized controlled trial designed to assess the effect of 12-week behavioral modification interventions on cardiovascular, metabolic, and functional recovery after acute takotsubo cardiomyopathy. We demonstrate that compared with standard care, physical exercise training and cognitive behavioral therapy resulted in improved resting cardiac energetics. They also improved physical exercise capacity measured from both cardiopulmonary exercise and 6-minute walk distance tests. Physical exercise training appears to also improve respiratory capacity (forced expiratory volume in 1 second, forced vital capacity, and maximum voluntary ventilation) compared with standard care. However, these objective improvements were dissociated from patient-reported heart failure symptoms, which did not change with trial interventions. We conclude that cardiac rehabilitation through behavioral modification has beneficial effects on cardiac metabolic and functional health in patients with takotsubo cardiomyopathy although these improvements did not translate into better symptom status.

Despite the rapid return of left ventricular ejection fraction to normal values in the weeks following acute presentation, patients with takotsubo cardiomyopathy demonstrate persistent structural, functional, and metabolic changes^2,3^ alongside decreased long-term survival.^1,19^ Given the current lack of evidence and absence of international guidelines or expert consensus recommendations, many cardiologists have implemented similar treatment strategies to treating acute myocardial infarction in terms of pharmacotherapy (β-blockers and angiotensin-converting enzyme inhibitors) and enrollment into cardiac rehabilitation programs. It is, therefore, a logical step to test whether exercise, which has good evidence for improved outcomes following acute myocardial infarction,^20^ has a role in the recovery of myocardial abnormalities or cardiovascular fitness after takotsubo cardiomyopathy. Given that takotsubo cardiomyopathy is a disease sitting at the interface between mental and physical health, we also considered an alternative psychological rehabilitation, in the form of cognitive behavioral therapy. Although this has much less supporting evidence in cardiovascular diseases, the psychological rehabilitation studies reported to date have been encouraging.^21^

In our study, the physical exercise training was delivered with a higher intensity than current cardiac rehabilitation programs, and patients also benefited from frequent interactions with health care professionals in both intervention arms. We showed that an exercise program appeared to be safe, well-tolerated, and readily accepted by patients early after the acute event. Cardiopulmonary exercise testing has been used to prescribe exercise training in patients with heart failure.^22^ However, we have previously demonstrated that a substantial proportion of patients with takotsubo cardiomyopathy, both in the acute phase and at medium-term follow-up, are unable to complete cardiopulmonary exercise testing. To ensure that our intervention cohort was as representative as possible, and to avoid introducing selection bias toward those able to undergo cardiopulmonary exercise testing, we chose not to use it to guide the prescribed exercise program. Thus, this study contributes, in a more general manner, to the evidence supporting the broad application of cardiac rehabilitation and exercise training in this population. In real-world practice, cardiopulmonary exercise testing is often not available, and requiring it for prescription could limit the reach and potential impact of such programs. It is likely that the success achieved in improving resting myocardial energetic recovery is reliant on both a direct effect of exercise and an increased level of confidence to pursue it. In the absence of any discernible differences in left ventricular ejection fraction or differences in resolution of myocardial edema between treatment groups, it is reasonable to infer that exercise training or increased physical activity may have a direct metabolic effect on the myocardium. Indeed, we have recently shown that physical training is a direct determinant of increased lipid turnover in the myocyte, and as free fatty acids are the main metabolic substrate utilized by cardiac myocytes, this merits further exploration in patients with takotsubo cardiomyopathy.^23^ In addition, there is a well-recognized myocardial and systemic inflammatory activation in acute takotsubo cardiomyopathy,^2^ which is responsible for the myocardial edema reported by several groups.^24^ Both human and animal models of stroke, heart failure, and myocardial infarction have indicated that exercise attenuates myocardial inflammation, and this is related to improved myocardial calcium handling and decreased inflammatory cytokine levels.^25–27^ As previously shown, there is defective myocardial calcium handling in acute takotsubo cardiomyopathy^28^ although its recovery in response to therapy has never been previously tested. Our study suggests that the metabolic and energetic impairment that occurs in acute takotsubo cardiomyopathy^2^ can be attenuated by exercise training.

We have previously demonstrated that patients with prior takotsubo cardiomyopathy have reduced maximal oxygen consumption due to cardiovascular limitation,^2^ compatible with a heart failure with preserved ejection fraction phenotype. In this work, we have demonstrated an improvement in the rate of oxygen consumption at peak exercise and 6-minute walk test distance from both physical exercise training and cognitive behavioral therapy. Studies into heart failure cohorts have demonstrated that a 6% increase in rate of oxygen consumption at peak exercise was associated with an 8% lower risk of heart failure hospitalization and a 7% lower risk of all-cause mortality,^29^ establishing assessment of exercise capacity as an important prognosticator. The 6-minute walk test is a sensitive and reproducible marker for performance and outcome^30^ and is a strong independent predictor of mortality and cardiovascular events in patients with chronic heart failure and ischemic heart disease.^31,32^

The cognitive behavioral therapy program applied in this study focused on behavioral changes leading to retraction from negative behaviors, improvements in mental well-being, and encouragement of physical activity. Cognitive behavioral therapy has been shown to help patients reframe unhelpful beliefs and behaviors, helping them to step out of the sick role, fostering a more active mindset. By challenging these patterns, cognitive behavioral therapy encourages gradual engagement in exercise and daily activities, supporting both physical and psychological recovery.^21^ Psychological stress is often the central trigger for takotsubo syndrome, and patients with takotsubo syndrome are more likely to have preexisting psychiatric illness. Patients with conditions such as anxiety and depression have been shown to have upregulated microRNA 16 and 26a, which, in rodent models, was associated with apical wall motion abnormalities in response to exogenous epinephrine.^33^ We and others have also demonstrated structural and functional brain differences between patients with acute takotsubo syndrome and sex-/age-matched healthy subjects.^34^ This supports the theory of the brain-heart axis with a predilection of the myocardium to develop takotsubo syndrome in response to stress. Here, we have demonstrated improvements in resting cardiac energetics and physical capacity measured from both cardiopulmonary exercise and 6-minute walk distance. Given that takotsubo is an acute systemic inflammatory condition, these findings may represent a direct effect on the brain-heart axis and a reduction in neuroinflammation. Larger studies with longer follow-up are required to assess the impact of these interventions on subsequent morbidity and mortality of patients with takotsubo cardiomyopathy.

We have also previously shown that like other heart failure syndromes, patients with takotsubo cardiomyopathy have lower forced expiratory volume in 1 second and forced vital capacity compared with age- and sex-matched healthy volunteers.^3^ The increase in forced expiratory volume in 1 second, forced vital capacity, and maximum voluntary ventilation following exercise training in this study represents an additional improvement and favors exercise training programs if feasible and acceptable to the patient. Lung function may have an important role in this condition as respiratory pathology, particularly chronic obstructive pulmonary disease, has a higher prevalence in takotsubo cardiomyopathy than in acute myocardial infarction and has been shown to represent a vulnerable phenotype with higher risk of in-hospital complications, long-term recurrence, and mortality.^35^

There was no difference in baseline left ventricular ejection fraction between the groups. This was derived from cardiac magnetic resonance imaging, which was performed between 2 and 21 days after their index event. As such, their left ventricular systolic function will have undergone a significant element of recovery already.

There were no demonstrable differences in the patient-reported symptom questionnaires between the 3 groups in our study. The Minnesota Living With Heart Failure Questionnaire is a commonly used measure of health-related quality of life and symptoms in heart failure. However, this scoring system was designed and validated for use in patients with reduced left ventricular ejection fraction in large populations.^36–38^ As we have demonstrated previously, patients with takotsubo cardiomyopathy have impaired left ventricular ejection fraction acutely, and while it subsequently recovers, a proportion of patients have a phenotype of heart failure with preserved ejection fraction.^5^ It is likely that this questionnaire lacks sufficient sensitivity to delineate change within the current population. The Minnesota Living With Heart Failure Questionnaire has limitations including factor structure, potential for bias based on social and mental health demographics, and a lack of clarity on its applicability to diverse heart failure patient populations.^36^ Takotsubo cardiomyopathy occurs due to complex interactions within the brain-heart axis, and it is possible that self-reported symptomatic and quality of life improvements are blunted by the composite psychosocial influence within this condition despite objective improvements in functional cardiorespiratory performance.^39^ As with any open-label trial, there is also the potential for bias, especially with self-reported symptoms. While this would tend to favor false positive rather than false negative findings, we cannot exclude the potential for reporting bias.

Interestingly, recent advances in machine learning have led to a risk prediction model (the InterTAK-ML model) for patients with takotsubo cardiomyopathy at risk of adverse short-term prognosis.^40^ Further research should focus on such models that can predict those likely to benefit from intervention.

### Limitations

The intensity with which our behavioral intervention programs were applied in this study may not be applicable in public health care systems and requires a cost-effectiveness assessment. There were no significant differences in the baseline characteristics. However, the standard of care group appeared to be older and had more hypertension and a higher proportion of physical triggers. This is also reflected in the baseline cardiopulmonary exercise testing variables in the standard of care group. Given the potential issue with power in clinical studies with small sample sizes, the potential impact on the measured outcome warrants further discussion. The baseline phosphocreatine/γATP ratio as the primary end point was similar between all 3 groups, suggesting comparable energetic impairment at baseline. It is possible, however, that those in the intervention groups could have potentially benefited more from exercise and cognitive behavioral therapy intervention through improved engagement, stronger baseline fitness, faster recovery, and better adaptability to the programs. In contrast, the control group showed an element of deconditioning in the exercise parameters, which may stem from either a lesser health status at baseline or lack of motivation/confidence due to lesser/no medical attention compared with the intervention groups. It is possible that these opposing trends may have contributed to the magnitude of the treatment effect. The results of this study should be interpreted within this context. Longer follow-up studies with larger study populations are required to establish the impact of exercise training and cognitive behavioral therapy on recurrent hospitalizations and mortality in patients with takotsubo cardiomyopathy.

### Conclusions

We have demonstrated improvements in left ventricular high-energy phosphate metabolism and cardiorespiratory functional capacity following behavioral modification after acute takotsubo cardiomyopathy. Larger studies to test the feasibility of implementing these rehabilitation strategies in health care systems are warranted.

## Article Information

### Acknowledgments

The authors acknowledge Prof Mark Petrie, Consultant Cardiologist, Glasgow; Dr Stuart Watkins, Consultant Cardiologist, Glasgow; Dr David Corcoran, Consultant Cardiologist, Glasgow; Prof Stephen Leslie, Consultant Cardiologist, Inverness; Dr David Carrick, Consultant Cardiologist, Hairmyres; Prof Chim Lang, Consultant Cardiologist, Dundee; and Dr Neil Anglim, Consultant Cardiologist, Dundee for identifying patients recruited in this study.

### Author Contributions

Dr Gamble recruited participants, scheduled, coordinated, and performed all clinical imaging investigations, analyzed all data, performed statistical analyses under the supervision of Dr Horgan (study statistician), and drafted this article. Dr Ross designed and developed the protocol for cardiac muscle spectroscopy. Dr Khan shared the delivery of physical exercise and cognitive behavioral therapy programs. L. Cheyne, A. Rudd, and J. Srivanasan helped with the investigations and reviewed and contributed to this article. Drs Hogg and Myint provided clinical input. Dr Newby obtained funding and reviewed and contributed to this article. Dr Williams created the living life to the full well-being program, oversaw its implementation, and trained the 2 cardiology fellows. Dr Gray created the exercise training intervention and oversaw its implementation. D. Dawson (principal investigator) designed the study, obtained funding and regulatory approvals, supervised the unfolding of the study and its analyses, and revised the draft writing. All authors have contributed to the manuscript writing.

### Sources of Funding

The British Health Foundation (BHF) grant PG/18/35/33786, Physical Exercise and Mental Wellbeing Rehabilitation for Acute Stress-Induced Takotsubo Cardiomyopathy: The PLEASE Trial, funded D. Dawson for the study and the salary of Dr Gamble. BHF grant FS/RTF/20/30009 funded D. Dawson for the salary of A. Rudd. Dr Newby is supported by the British Heart Foundation (grants CH/09/002, RG/F/22/110093, and RE/24/130012).

### Disclosures

None.

### Supplemental Material

Supplemental Methods

Table S1

Figure S1

## Supplementary Material
