# Supplementary material for: Physical Exercise or Cognitive Behavioral Therapy for Takotsubo Cardiomyopathy: A Randomized Controlled Trial
Source: Circ Heart Fail. 2025 Dec 3;19(3):e013229. doi: 10.1161/CIRCHEARTFAILURE.125.013229 (PMC12986043; doi:10.1161/CIRCHEARTFAILURE.125.013229)
Supplement: Supplementary file 1 [file hhf-19-e013229-s001.pdf]

Supplemental Material

Supplemental Methods:

For **^{31}P-Magnetic Resonance Cardiac Muscle Spectroscopy**, the cardiac acquisition B_0 shimming was performed over the entire heart using a projection-based method. Immediately prior to the ^{31}P acquisition the field homogeneity was manually assessed by measuring the linewidth from a ^{1}H point resolved spectroscopy voxel placed in the interventricular septum. ^{31}P- Magnetic Resonance Spectroscopy signal localisation was performed using 1D-Chemical Shift Imaging using adiabatic excitation with 24 phase encoding steps positioned parallel to the chest wall with phase-encoding step size 18.75 mm, 4 averages, 2048 sample points and a sampling bandwidth of 3 kHz. Saturation bands were placed over the chest wall and the liver to minimise spectral contamination. All ^{31}P-cardiac spectroscopy acquisitions were ECG-gated, triggered to mid-late diastole, with a typical repetition time of ≥10 seconds. All spectroscopy data were analysed in JMRUi 5.2 using the advanced method for accurate, robust, and efficient spectral fitting (AMARES) algorithm [2] using spectra extracted from voxels containing the interventricular septum. Cramér-Rao standard deviations of all peaks were calculated, and only those <20% were accepted for inclusion in the analysis.

For **cardiac magnetic resonance imaging**, the imaging protocol comprised the following: 1) balanced steady state free precession cine imaging in long axes and a full ventricular short axis stack (echo time (TE) 1.49 ms, repetition time (TR) 3 ms, field of view (FOV) 250 mm foot – head (FH) x 250 mm anterior to posterior (AP) x 107 mm right to left (RL), voxel size 1.8 mm FH x 1.8 mm AP, 45° flip angle, slice thickness/gap 7/3 mm with image acceleration using Philips compressed sense with 30 cardiac phases); 2) balanced gradient echo readout with a Modified Look-Locker Inversion recovery (MOLLI, 5[3]3) scheme native T1 mapping (TE 0.94 ms, TR 2.0 ms, field of view (FOV) 300 mm FH x 300 mm AP x 47 mm

RL, voxel size 2 mm FH x 2 mm AP, 20° flip angle, slice thickness/gap 10/8.5); 3) Gradient And Spin Echo readout T2 mapping (TE 0.75 ms, TR 1.92 ms, field of view (FOV) 300 mm FH x 300 mm AP x 48 mm RL, voxel size 2 mm FH x 2 mm AP, 20° flip angle, slice thickness/gap 10/9); 4) early and late post gadolinium enhancement (0.1 mmol kg^{-1} gadolinium diethylenetriamine-pentacetate, Gadovist; Bayer, Leverkusen, Germany) using a spoiled gradient echo inversion recovery sequence (TE 3.0 ms, TR 6.1 ms, FOV 320 mm FH x 356 mm AP x 108 mm RL, voxel size 1.8 mm FH x 2.2 mm AP, 25° flip angle, slice thickness/gap 8/2 mm) with swapping of the phase-encoding direction to exclude artefact, matching the cine images and 5) post-contrast T1 enhanced balanced gradient echo readout with a MOLLI 5[3]3 scheme acquired at exactly 10 min after contrast administration (TE 0.94 ms, TR 2 ms, FOV 300 mm FH x 300 mm AP x 47 mm RL, voxel size 2 mm FH x 2 mm AP, 20° flip angle, slice thickness/gap 10/8.5 mm). Endocardial and epicardial borders of the motion corrected parametric maps were traced manually and set 10% inwardly to avoid blood on the endocardial side and fat or extracardiac structures on the epicardial side. Epicardial, endocardial and papillary muscle borders were automatically detected and then adjusted manually to ensure they were accurately traced. A minority of artefactual images without clear epicardial and endocardial borders were excluded. All parametric images were collected using the Philips implementation of a motion corrected elastic image registration technique [1].

For **2D-echocardiography**, three cine loops in each of the standard recommended British Society of Echocardiography views (parasternal long-axis, short-axis, and apical four-, three-, and two-chamber views) were obtained at a frame rate of at least 85 Hz. Together with colour and continuous/pulsed wave doppler examination a full dataset was stored for offline analysis. Global longitudinal strain (GLS) was obtained after manually adjusting the automatic detection of the epicardial and endocardial borders in each of the three long axis views.

Supplemental Figures

Figure S1: Consort Diagram: Study Flowchart

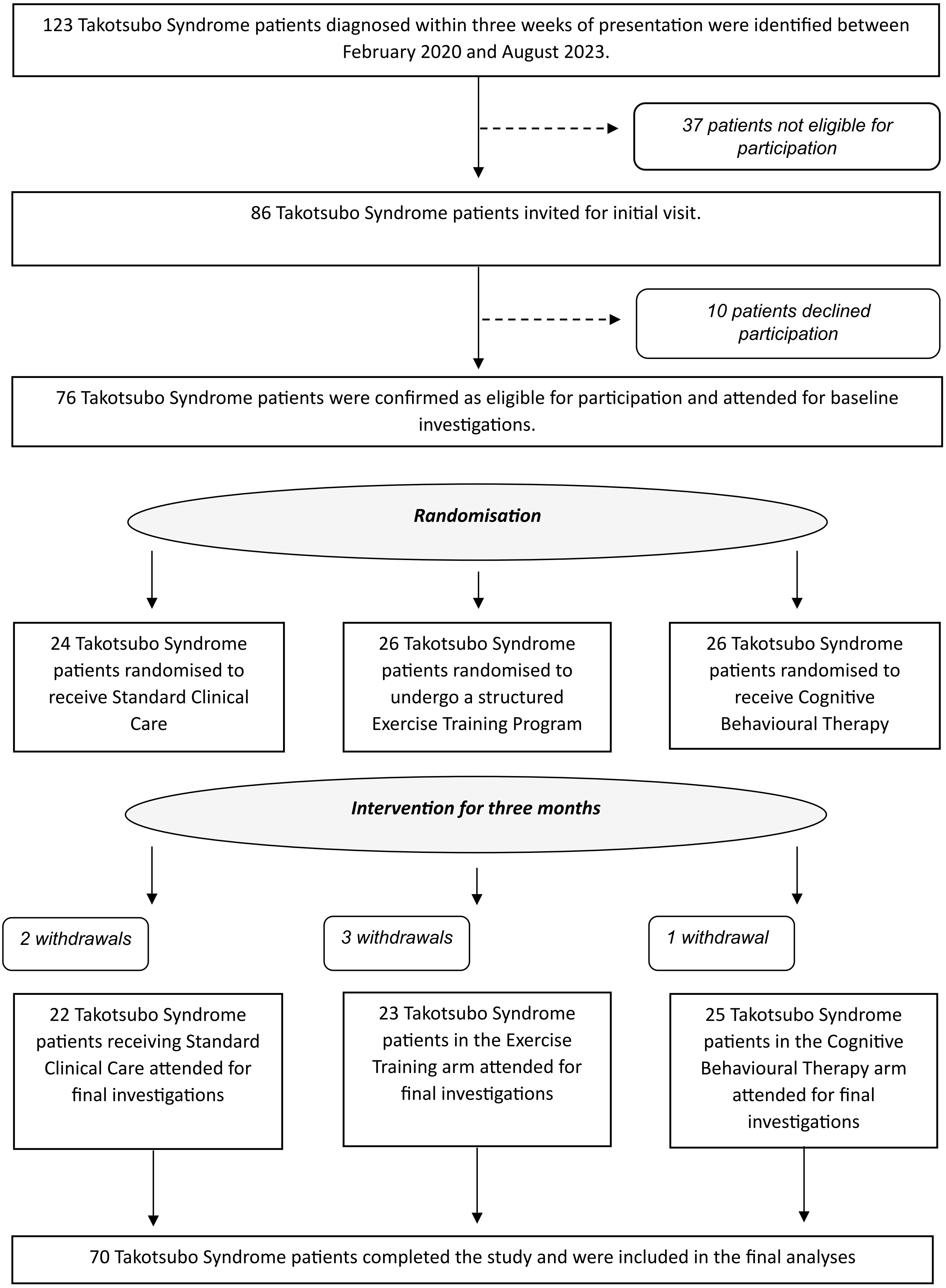

Supplemental Tables

	**Effect size**	**Standard Error**	**95% confidence interval**	**P value**	**Levene's test of equality of error variances**	**Lack of Fit Test**
PCr/γATP ratio						
Age	0.005	0.008	-0.012 – 0.022	0.573	0.972	0.600
Gender	0.052	0.223	-0.403 – 0.507	0.816		
COVID	0.293	0.245	- 0.207 – 0.792	0.241		
Indexed left ventricular end diastolic volume (ml/m^2)						
Age	-0.281	0.125	-0.532 - -0.029	0.030	0.107	0.474
Gender	-7.362	3.257	-13.901 - -0.824	0.028		
COVID	-0.243	3.798	-7.869 – 7.382	0.949		
Indexed left ventricular end systolic volume (ml/m^2)						
Age	-0.164	0.115	-0.395 – 0.066	0.159	0.972	0.068
Gender	-4.850	2.989	-10.851 – 1.151	0.111		
COVID	1.082	3.486	- 5.919 – 8.081	0.757		
Indexed left ventricular mass (g/m^2)						
Age	-0.227	0.183	-0.593 – 0.140	0.220	0.135	0.211
Gender	0.749	4.748	-8.783 – 10.280	0.875		
COVID	1.453	5.537	-9.664 – 12.569	0.794		
Ejection Fraction (%)						
Age	-0.015	0.158	-0.332 – 0.302	0.925	0.312	0.877
Gender	3.107	4.153	-5.227 – 11.441	0.458		
COVID	-0.224	4.851	-9.958 – 9.510	0.963		
Left ventricular T1 mapping (ms)						
Age	-2.783	1.056	-4.900 - -0.665	0.011	0.762	0.128
Gender	-4.986	-0.165	-65.770 – 55.797	0.870		
COVID	-24.904	41.821	-108.787 – 58.979	0.554		
Left ventricular T2 mapping (ms)						
Age	-0.43	0.090	-0.223 – 0.138	0.635	0.642	0.463
Gender	-0.033	2.670	- 5.408 – 5.342	0.990		
COVID	-	-	-	-		

METs						
Age	0.005	0.017	-0.030 – 0.40	0.768	0.592	0.928
Gender	-0.032	0.547	-1.143 – 1.079	0.954		
COVID	-0.034	0.479	-1.007 – 0.938	0.944		
FEV1 (L)						
Age	-.006	0.003	-0.012 - -0.001	0.032	0.960	0.418
Gender	0.137	0.092	- 0.049 – 0.323	0.144		
COVID	0.015	0.080	- 0.148 – 0.178	0.574		
FVC (L)						
Age	-0.004	0.004	-0.011 – 0.003	0.280	0.610	0.234
Gender	0.228	0.112	0.455 – 0.0103	0.049		
COVID	0.043	0.098	-0.157 – 0.242	0.668		
Breathing Reserve (L/min)						
Age	0.054	0.190	-0.332 – 0.440	0.776	0.802	0.542
Gender	1.720	6.013	-10.486 – 13.926	0.777		
COVID	1.189	5.261	-9.492 – 11.869	0.823		
Maximum voluntary ventilation (l/min)						
Age	-0.257	0.117	-0.495 – -0.019	0.035	0.951	0.495
Gender	5.592	3.715	-1.950 – 13.134	0.141		
COVID	0.681	3.250	-5.918 – 7.280	0.835		
Peak VO_2 (mL/min/kg)						
Age	0.017	0.055	-0.095 – 0.129	0.763	0.495	0.535
Gender	-1.194	1.743	-4.734 – 2.345	0.498		
COVID	-0.893	1.525	-3.989 – 2.204	0.562		
VE/VCO_2 slope						
Age	-0.104	0.126	-0.363 – 0.155	0.416	0.874	0.450
Gender	3.665	2.995	-2.491 – 9.820	0.232		
COVID	-3.465	2.812	-9.246 – 2.316	0.964		
Six minute walk distance (m)						
Age	-2.008	1.347	-4.743 – 0.727	0.145	0.606	0.783
Gender	52.636	43.291	-35.250 – 140.522	0.232		
COVID	-15.129	38.211	-92.702 – 62.444	0.695		
Global Longitudinal Strain (%)						
Age	-0.067	0.094	-0.254 – 0.121	0.480	0.721	0.780
Gender	2.142	2.800	- 3.485 – 7.769	0.448		
COVID	-0.685	3.344	-7.406 – 6.036	0.839		
B-type natriuretic peptide (pg/mL) - Rest						

Age	-0.852	2.051	-4.983 - 3.279	0.680	0.026	0.971
Gender	-86.773	69.055	-225.857 – 52.311	0.215		
COVID	20.317	59.519	-99.561 – 140.194	0.734		
B-type natriuretic peptide (pg/mL) – peak exercise						
Age	-2.374	3.460	-9.422 – 4.673	0.497	0.268	0.998
Gender	-115.402	101.343	-321.831 – 91.027	0.263		
COVID	76.803	88.045	-102.540 – 256.146	0.390		
B-type natriuretic peptide (pg/mL) – 15-minute post-recovery						
Age	-0.988	2.962	-7.037 – 5.062	0.741	0.517	0.932
Gender	-107.656	86.301	-283.906 – 68.594	0.222		
COVID	48.757	74.358	-103.102 – 200.616	0.517		
MLHFQ - Total						
Age	0.500	0.361	-0.226 – 1.225	0.173	0.176	0.036
Gender	4.059	12.116	-20.276 – 28.395	0.739		
COVID	2.269	10.872	-19.568 – 24.105	0.836		
MLHFQ – Physical domain						
Age	3.151	5.173	-0.084 – 0.606	0.135	0.280	0.043
Gender	0.642	5.765	-10.938 – 12.222	0.912		
COVID	3.151	5.173	-7.240 – 13.541	0.545		
MLHFQ – Emotional domain						
Age	0.166	0.110	-0.055 – 0.388	0.138	0.128	0.134
Gender	1.441	3.698	-5.988 – 8.869	0.699		
COVID	-1.223	3.319	-7.888 – 5.443	0.714		

Table S1: Regression table for paired ANCOVA including effect size, standard errors, 95% confidence intervals, and p-values for each covariate, as well as overall model diagnostics. Model covariates were age, gender and COVID interruption. PCr/γATP ratio - phosphocreatine/gamma adenosine triphosphate. RER - respiratory exchange ratio. MET – metabolic rate. FEV1 – forced expiratory volume in 1 second. FVC – forced vital capacity. Peak VO2 - rate of oxygen consumption at peak exercise. VE/VCO_2 - slope of the minute ventilation/carbon dioxide production relationship. MLHFQ - Minnesota living with heart failure questionnaire. For cardiopulmonary exercise testing only those with RER > 1.1 at both baseline and follow-up were included.
